# Supplementary material for: A Critical View of the Application of the APEX Software (Aqueous Photochemistry of Environmentally-Occurring Xenobiotics) to Predict Photoreaction Kinetics in Surface Freshwaters
Source: Molecules. 2019 Dec 18;25(1):9. doi: 10.3390/molecules25010009 (PMC7017383; doi:10.3390/molecules25010009)
Supplement: Supplementary file 1 [file molecules-25-00009-s001.zip › 3.2.4_gcc-4.4.0.zip!/3.2.4_gcc-4.4.0/3.2.4_gcc-4.4.0/share/octave/packages/simp-1.1.0/doc/simp.pdf]

Single Interval Mathematics Package for Octave

Simone Pernice

January 6, 2009

Contents

1 Author and version 4

2 Preface 4

3 Introduction 5

4 Intervals 6

5 Issues with interval computation 8
- 5.1 Variables independence . . . 8
- 5.2 Hyper-cube Overlapping . . . 8

6 Introduction on how the package works 8
- 6.1 How to run SIMP in Octave . . . 8
  - 6.1.1 Install SIMP . . . 9
  - 6.1.2 Remove SIMP . . . 9
- 6.2 Some example . . . 9

7 Functions 11
- 7.1 Intervals . . . 11
- 7.2 valtol100ToInt (v1, ptol, ntol) . . . 11
- 7.3 intToTol (v1) . . . 12
- 7.4 intToTol100 (v1) . . . 12
- 7.5 intToVal (v1) . . . 12
- 7.6 engFormInt (v1) . . . 12
- 7.7 intFormInt (string) . . . 13
- 7.8 addInt (v1, v2, v3, ...) . . . 13
- 7.9 negateInt (v1) . . . 13
- 7.10 subInt (v1, v2, v3, ...) . . . 13
- 7.11 mulInt (v1, v2, v3, ...) . . . 13
- 7.12 invertInt (v1) . . . 13
- 7.13 invertAddInt (v1, v2, v3, ...) . . . 14
- 7.14 divInt (v1, v2, v3, ...) . . . 14
- 7.15 powerInt (v1, n) . . . 14
- 7.16 sqrInt (v1) . . . 14
- 7.17 sqrtInt (v1) . . . 14
- 7.18 sqrAddInt (v1, v2, v3, ...) . . . 14
- 7.19 dB10ToLinInt (v1) . . . 14
- 7.20 linToDB10Int (v1) . . . 14
- 7.21 dB20ToLinInt (v1) . . . 14
- 7.22 linToDB20Int (v1) . . . 14
- 7.23 linSpaceTol100Int (begin, end, numberOfIntervals, tolerance) . . . 15
- 7.24 logSpaceTol100Int (begin, end, numberOfIntervals, tolerance) . . . 15
- 7.25 monotonicFunctionInt (f, x) . . . 15
- 7.26 functionInt (f, x, nOfPoint) . . . 15

7.27 plotInt (x, y) 15
7.28 errorBarInt (x, y) 15

8 Biography **16**

1 Author and version

I am Simone Pernice the writer of the SIMP package. If you find any bug or you want to add features, you can contact me at pernice@libero.it. You can find further details on my web site: simonepernice.freehostia.com

List of versions of this document:

- Version 1.0, Turin 14th December 2008, initial draft
- Version 1.1, Palermo 2nd January 2009, few fixes to the documentation and added the new function intFormInt

2 Preface

I am an electrical engineer, I worked both on hardware and software fields. Designing an electrical circuit is quite complex because the characteristics of the components involved have huge tolerance some about 5%, while others 50%. The target is to have the circuit working in the worst case of the components and of the environment! That involves to compute several times the same equation putting inside the worst or best case values. Some time ago I was designing a simple circuit when I though: "What if I use intervals instead of numbers? I may get the best and worst case in just one step!". Then I looked on the Internet for Interval Mathematics: I discovered it was invented in the '50s [1]. Although its not very spread it is a powerful tool: a calculator with finite precision can be used to compute calculus requiring infinite precision because the solution is a range where the actual value lay.

Therefore I looked for an open source calculator able to manage intervals instead of real numbers. Unfortunately I was not able to find anything. There are just few libraries for the C++ language and packages for proprietary software. I was thinking to develop a calculator on my own, but it would have required a lot of effort and long time. Then I discovered Octave[3]. By the way Octave is a great tool that can be used by every engineer for its daily work. After a look at Octave's tutorial [4] I decided to write a package to work on intervals in Octave. It was the right choice, because it took just three days to make it while building a new program from scratch would have taken weeks. The package extends the basic arithmetic functions to single interval mathematics, it is called SIMP. As far as I know it is not possible to overload Octave operators with m script files, therefore I made new functions working on matrix to extend the basic operations to intervals. In Octave it is possible to write the first few chars of a function and then push the TAB key to get the list of the functions compatible. Moreover it is really simple to build intermediate variables. That makes not too long using the SIMP functions for standard calculations.

Section 3 shows why numbers are not the best tool when computing real world entities. Section 4 explains what is an interval and how a function can be extended to work on intervals. Section 5 explains some issues to be aware when making interval calculations, which are not present in the number computation.

Section 6 shows some examples to explain how the SIMP package can be used. Eventually the Section 7 provides the detailed list of functions available.

3 Introduction

SIMP is a simple interval calculator. Intervals can be used instead of floating point number to automatically take into account the tolerance of the values used in calculation. All functions can be extended to interval: therefore it is possible to apply all the functions to intervals to get the final result as an interval. If you understand what intervals are, you can go directly to Section 6 to read some examples and then to Section 7 to see all the functions available.

Every day we need to compute the result of a lot of simple mathematical equations. For example the cost of the apples bought at the supermarket is given by the apple cost per kilo times the number of kilos bought: $applePrice = appleCostPerKilo \times kilosOfAppleBought$.

When we need the result of those mathematical expressions, we put the values on the right side of the equation and we got its result on the left side. Well, we usually put wrong numbers on the right side and therefore there is no doubt we get wrong results. There are a lot of reasons why we put wrong values, below some of them follows:

1. Most of the values are measured, therefore they are known within a given tolerance (looking for accuracy and precision on Wikipedia will provide interesting information on that matter);

2. Some values have an infinite number of digits after the decimal point (real number), like for π (the ratio between a circumference and its diameter);

3. Some values change with time or samples (or whatever), like the weight of a person (which can change of 5% during a day) or the current gain of a BJT (which can change of 50% on the samples of the same series);

4. Some value are estimation or guess: something like between a minimum and a maximum.

For example if a pipe brakes and you want to buy a new one you need its diameter. If you do not have a caliber, you may measure its circumference and divide it by π (3.1415...):$diameter = \frac{circumference}{\pi}$.

Here there are two errors: the circumference is known within the tolerance given by your meter, moreover π has an infinite number of digits while only few of them can be used in the operation. You may think the error is negligible, the result is enough accurate to buy a new pipe in a hardware shop. However the not infinite accuracy of those operation avoid the use of computers as automatic theorem demonstration tools and so on... This kind of issue is quite common on engineer design. What engineers do is to be sure their design will work in the worst case or in most of the cases (usually more than 99.9%).

Here a simple example follows. Let us say you want to repaint the walls of your living room completely messed up by your children. You need to compute how many paint cans you need to buy. The equation is quite simple:

$$paintCans = \frac{2 \times (roomWidth + roomLength) \times roomHeight}{paintLitersPerCan \times paintEfficiency} \quad (1)$$

where paintEfficiency is how many square meters of surface can be painted with a liter of paint. The problem here is that usually we do not have a long enough meter to measure the room width and length, it is much simpler to count the number of steps to go through it (1 step is about 1m, let us say from 0.9 to 1.1m). Moreover the paint provider usually declare a paint efficiency range. Let us put below the data:

- roomWidth = 6 (from 5.4m to 6.6m)
- steps roomLength = 4 (from 3.6m to 4.4m)
- steps roomHeight = 3 (it is assumed correct because its tolerance is much smaller than the other pieces)
- meters paintEfficiency = from 0.7 to 1.3 square meters per liter (1 liter per square meter in average)
- paintLitersPerCan = 40 (it is assumed correct because its tolerance is much smaller than the other pieces)

To compute the average result just put average values in (1). We get: $paintCans = \frac{2 \times (6+4) \times 3}{40 \times 1} = 1.5$

paint cans, which means two unless you are able to buy just half of a can.

Are you satisfied with that result? I am not. What if I have underestimated something? As every engineer I would check what happens in the worst case, which means 1.1m step and efficiency of just 0.8. Again just substituting those values in (1), we get: $paintCans = \frac{2 \times (6.6+4.4) \times 3}{40 \times 0.7} = 2.36$. That is really interesting: in the worst case I would miss 0.36 cans, it makes sense to buy three cans to avoid to go back to the hardware shop to buy one more (in the worst case).

More happy with the result now? I am not completely satisfied, I am asking myself: what if in the best case I need just 1 can? In that case probably I need more accurate data because the result range would be too wide. Eventually from (1) we get: $paintCans = \frac{2 \times (5.4+3.6) \times 3}{40 \times 1.3} = 1.04$. Which means two cans. I am satisfied, I have to buy at least two cans, but probably I may need one more. In the next paragraph you will see how to do all this stuff in one step using Octave and SIMP!

4 Intervals

As you saw in the example above a lot of computations are required to get an idea of the range where the result of the equation lay. To get the worst case,

you need to think carefully at the equation because some time you need to put the higher value (like for steps in (1)) while other times the smallest (like for efficiency in (1)) to get the worst case and vice versa for the best. There is a much simpler way to work with that issue. You can use intervals instead of number[1, 2]. An interval is:

$$[a1, a2] = \{\forall x \in \mathbb{R} \mid \mathit{a1} \leqslant x \leqslant \mathit{a2}\}\,; a1, a2 \in \mathbb{R} \cup \{-\infty, +\infty\}\,; \mathit{a1} \leqslant \mathit{a2}; \quad (2)$$

In few words, [a, b] is the set of real numbers among a and b. Please note that intervals suit perfectly in all the cases where numbers do not fit (some were showed in the Section 3).

Moreover when it is correct to use a number, it is possible to use a degenerate interval like [a, a]. That means intervals can replace real numbers in every computation.

It is possible also to define functions that work on intervals instead of numbers:

$$f\left([a1, a2], [b1, b2], ...\right) = \{\forall f(a, b, ...) \in \mathbb{R} \mid \exists a \in [a1, a2], b \in [b1, b2], ...\} \quad (3)$$

Please note, with that definition it is possible to extend every function (like addition, multiplication, square, square root, ...) to work on intervals.

Note also sometime a function may generate several intervals as result. As an example consider the following division: $[4, 4]/[-2, 2] = [-\infty, -2] \cup [2, +\infty]$, it generates two interval as result. However SIMP works only on single interval function, for that reason if you try to divide for a interval containing 0 in SIMP, you will get an error.

Eventually note that usually computing the interval result of a function applied to intervals requires a lot of steps. Therefore sometime we may be satisfied to find, in a short time, a bigger interval containing the smallest interval where the solution must lay.

In the example above you would have:

- roomWidth = [5.4, 6.6] meters
- roomLength = [3.6, 4.4] meters
- roomHeight = 3 = [3, 3] meters
- paintEfficiency = [0.7, 1.3] square meters per liter
- paintLitersPerCan = 40 = [40, 40] liters per can

Now you have just to compute the equation once with extended function to get [1.04, 2.36] cans. SIMP, the Octave package I developed, extends all the basic mathematical functions and it provides functions to extend all other available Octave functions.

5 Issues with interval computation

There are few issues you need to know about interval computations because they do not happen in standard mathematics[2].

5.1 Variables independence

All the variables you use to make a computation are independent from each other, although they are the same instance of variable for you. For example the perimeter of a rectangle (which was also used in a piece of the equation 1) can be written in several ways. If the dimensions of the rectangle are b and h we can write:

$$perimeter = 2(h + b) \tag{4}$$

$$perimeter = h + h + b + b \tag{5}$$

In standard mathematics they are the same, while in interval mathematics we have the interval $equation(4) \subsetneq equation(5)$. The reason is that every interval that appears in the equation is not dependent from each other, therefore in equation 5 is like having four different dimensions (h, h', b and b').

What is important to remember is that: *Every variable should appear just once in the equation (if possible) in order to get the smallest interval possible as result.*

5.2 Hyper-cube Overlapping

The result of a set of simultaneous equations involving intervals is *always* an hyper-cube. For example if we have two simultaneous equations in the unknowns x and y the result will be a couple of intervals which draw a rectangle on the Cartesian plane. However the solution of a couple of simultaneous equations may be smaller, for example a segment on the Cartesian plane. In that case the rectangle will contain the segment. There is no way to solve that, just remember the solution is usually a super set of the actual one.

6 Introduction on how the package works

6.1 How to run SIMP in Octave

Octave [3] is a powerful numeric calculator, which is what is required for design. It is open source and freely available on a lot of platform. I suggest to play with Octave to understand its basic functions[4, 5] before using SIMP package.

6.1.1 Install SIMP

First of all you have to install the SIMP package in Octave. To do that just run Octave from the directory where was downloaded the package and execute the following command:

- pkg install simp.tar.gz

From the next execution of Octave, it will automatically load the new functions. Therefore SIMP functions will be available as the standard Octave ones.

6.1.2 Remove SIMP

If for some reason you want to remove the SIMP package, you can write:

- pkg uninstall simp.tar.gz

From the next execution of Octave all the SIMP functions will not be available.

6.2 Some example

SIMP works on matrix, interpreted as intervals. The basic interval is: [min, max], which in Octave is a row vector made by two elements. The added functions work on those matrixes. For example to add the intervals [5.4, 6.6] and [3.6, 4.4] it is possible to write:

- addInt ([5.4, 6.6], [3.6, 4.4])

$>$ ans = [9, 11]

addInt stands for add intervals. All SIMP functions end with the word "Int". It is possible to use scalar number instead of intervals. Every scalar is computed like: $s = [s, s]$. Therefore the following computations give the same result:

- addInt ([5.4, 6.6], 3.6)

$>$ ans = [9, 10.2]

- addInt ([5.4, 6.6], [3.6, 3.6])

$>$ ans = [9, 10.2]

Octave supports variable declaration, therefore it is possible to write:

- roomWidth = [5.4, 6.6];
- roomLength =[3.6, 4.4];
- addInt (roomWidth, roomLength)

> ans = [9, 11]

Now we know enough Octave syntax to compute the equation 1:

- roomHeight = 3;
- paintEfficiency = [0.7, 1.3];
- paintLitersPerCan = 40;
- divInt(mulInt(2, addInt (roomWidth, roomLength), roomHeight), mulInt(paintLitersPerCan, paintEfficiency))

> ans = [1.0385, 2.3571]

Sometime the intervals are expressed in terms of tolerance. The electronic resistors are sold in several tolerance classes: 1%, 2%, 5%. There is a SIMP function to easily make an interval from a tolerance. For example a resistor of 10KOhms with 5% of tolerance can be converted in an interval:

- tollToInt (10000, 5)

> ans = [9500, 10500]

If the tolerance is not symmetric, for example +5% and -10%:

- tollToInt (10000, 5, -10)

> ans = [9000, 10500]

It is also possible to convert values from engineer notation to interval:

- intFormInt([“10K+-5%”)

> ans = [9500, 10500]

- intFormInt([“10K+5%-10%”)

> ans = [9000, 10500]

All the computations are done among intervals in SIMP. Therefore there is a SIMP function to convert an interval in engineer notation (in the hypothesis that the tolerance is symmetrical):

- engFormInt([9500, 10500])

> ans = “10K+-5%”

Eventually those functions work on vectors of intervals which may be useful to compute functions. It is possible to compute operation on vectors of the same size (element by element) or scalar by vector. In that case the scalar is expanded to a vector of the same size of the others. That is really useful for the functions where a x vector can be used to compute a y in just one time.

- vect1 = [1 2;3 4;5 6]

> vect1 =

> 1 2

> 3 4

> 5 6

- addInt (vect1, vect1, 1)

> vect1 =

> 3 5

> 7 9

> 11 13

As you can see SIMP can be used just like a calculator. The basic operations are applied on intervals instead of scalar. That makes really simple to evaluate equations where some data is uncertain.

7 Functions

This section describes the functions provided in the SIMP package.

7.1 Intervals

All the SIMP functions work on vector of intervals as input.

A vector of intervals is a matrix with two columns and many rows: every row describes an interval. The matrix is composed by real numbers.

An interval is a degenerate vector with two columns and only one row. The first value is the interval minimum, the second is the interval maximum. Therefore [1.1, 2.1] is an interval with 1.1 as lower boundary and 2.1 as upper boundary.

A real number is meant as a case of degenerate interval with maximum and minimum values coincident. Therefore 5 is like [5, 5].

It is useful working on vector because the same operation can be applied to several intervals in one step. All the vectors involved must have the same number of rows (intervals), with the exception of scalar and single interval. They are automatically expanded to the number of rows (intervals) that matches the other inputs.

7.2 valtol100ToInt (v1, ptol, ntol)

valtol100ToInt produces an interval with center value given by the scalar v1, and positive and negative tolerance express in percentage given by ptol and ntol. ntol is optional, if ntol is not present it is assumed equal to -ptol.

7.3 intToTol (v1)

intToTol produces the tolerance of the vector of interval v1, in the hypothesis that the actual value is in the average point between minimum and maximum.

7.4 intToTol100 (v1)

intToTol100 produces the tolerance (expressed in percentage) of the vector of interval v1, in the hypothesis that the actual value is in the average point between minimum and maximum.

7.5 intToVal (v1)

intToVal produces the central value of the vector of intervals v1, in the hypothesis that the actual value is in the average point.

7.6 engFormInt (v1)

engFormInt returns a vector of strings representing the intervals in the vector v1 in engineer notation. As value it returns the average between minimum and maximum which means symmetric tolerance.

The engineer notation is obtained finding the exponent (multiple of three) so that the mantissa is between 1 and 1000, then the exponent is converted into a letter as per the following list:

- Y = 10^{24}
- Z = 10^{21}
- E = 10^{18}
- P = 10^{15}
- T = 10^{12}
- G = 10^{9}
- M = 10^{6}
- K = 10^{3}
- nothing = 10^{0}
- m = 10^{-3}
- u = 10^{-6}
- n = 10^{-9}
- p = 10^{-12}

- f = 10^{-15}
- a = 10^{-18}
- z = 10^{-21}
- y = 10^{-24}

Eventually the tolerance is added in form of percentage: "+-" tolerance in percentage "%". Please note that the tolerance is symmetric which is not always the actual case. For example engFormInt([9500, 10500]) = "10K+-10%".

Note that with other functions (7.2) it is possible to define intervals with not symmetric tolerance, they will not be converted properly back by 7.6.

7.7 intFormInt (string)

intFormInt returns a vector of intervals given a vector of strings (or a single string) representing the number in engineer notation. See 7.6 for details on engineer notation. For example intFormInt("10K+-10%") = [9500, 10500].

Although it is not orthogonal to 7.6, it evaluates also expression with not symmetric tolerance: 10K+5%-10% is converted to [9000, 10500]. That was done because not symmetric tolerance is quite common in practice.

7.8 addInt (v1, v2, v3, ...)

addInt adds the given vector of intervals. It returns $v1 + v2 + v3 + \dots$. They must have the same number of rows or be single interval or be scalars. At least two vectors are required.

7.9 negateInt (v1)

negateInt negates the given vector of intervals. It returns $-v1$.

7.10 subInt (v1, v2, v3, ...)

subInt subtracts the given vector of intervals. It returns $v1 - v2 - v3 - \dots$. They must have the same number of rows or be single interval or be scalar. At least two vectors are required.

7.11 mulInt (v1, v2, v3, ...)

mulInt multiplies the given vector of intervals. It returns $v1 \cdot v2 \cdot v3 \cdot \dots$. They must have the same number of rows or be single interval or be scalar. At least two vectors are required.

7.12 invertInt (v1)

invertInt inverts the given vector of intervals. It returns $\frac{1}{v1}$.

7.13 invertAddInt (v1, v2, v3, ...)

invertAddInt adds the inverse of the given vector of intervals and then inverts again the result. It returns $\frac{1}{\frac{1}{v1}+\frac{1}{v2}+\frac{1}{v3}+\ldots}$. They must have the same number of rows or be single interval or be scalar. At least two vectors are required.

7.14 divInt (v1, v2, v3, ...)

divInt divides the given vector of intervals. It returns $\frac{\frac{v1}{v2}}{v3}\ldots$. They must have the same number of rows or be single interval or be scalar. The vectors v2, v3, ... must not include 0. At least two vectors are required.

7.15 powerInt (v1, n)

powerInt rise the given vector of intervals to the power of n. It returns $v1^n$.

7.16 sqrInt (v1)

sqrInt squares the given vector of intervals. It returns $v1^2$.

7.17 sqrtInt (v1)

sqrtInt square roots the given vector of intervals. It returns $\sqrt{v1}$.

7.18 sqrAddInt (v1, v2, v3, ...)

addSqrInt adds the square of the given vector of intervals and then it squares root the result. It returns $\sqrt{v1^2 + v2^2 + v3^2 + \ldots}$. They must have the same number of rows or be single interval or be scalar. At least two vectors are required.

7.19 dB10ToLinInt (v1)

db10ToLinInt rises 10 to the power of v1 tens. It returns $10^{\frac{v1}{10}}$.

7.20 linToDB10Int (v1)

linToDB10Int produces 10 times the logarithm of v1. It returns $10log_{10}(v1)$.

7.21 dB20ToLinInt (v1)

db10ToLinInt rises 10 to the power of v1 twenties. It returns $10^{\frac{v1}{20}}$.

7.22 linToDB20Int (v1)

linToDB10Int produces 20 times the logarithm of v1. It returns $20log_{10}(v1)$.

7.23 linSpaceTol100Int (begin, end, numberOfIntervals, tolerance)

linSpaceTol100Int produces a vector of numberOfIntervals intervals equally spaced between begin and end, with the given tolerance.

7.24 logSpaceTol100Int (begin, end, numberOfIntervals, tolerance)

linSpaceTol100Int produces a vector of numberOfIntervals intervals equally spaced between 10^{begin} and 10^{end}, with the given tolerance.

7.25 monotonicFunctionInt (f, x)

monotonicFunctionInt produces the y vector of interval obtained applying the monotonic function f to the vector of interval x. If f is not monotonic, y may be wrong.

7.26 functionInt (f, x, nOfPoint)

functionInt try to produces the y vector of interval obtained applying the function f to the vector of interval x. The function is checked for monotonicity on the given number of point. nOfPoint is optional, default is 10.

7.27 plotInt (x, y)

plotInt plots the interval of vector y respect to the interval of vector x. Those curves, for every x interval center point, goes through ymin and ymax. x and y must have the same rows.

7.28 errorBarInt (x, y)

For every x center point, plots the y tolerance.

8 Biography

References

[1] http://en.wikipedia.org/wiki/Interval_arithmetic

[2] http://www.cs.utep.edu/interval-comp/hayes.pdf

[3] http://www.octave.org

[4] http://www-mdp.eng.cam.ac.uk/web/CD/engapps/octave/octavetut.pdf

[5] http://www.gnu.org/software/octave/docs.html
